# Observer agreement for small bowel ultrasound in Crohn’s disease: results from the METRIC trial

**DOI:** 10.1007/s00261-020-02405-w

**Published:** 2020-02-10

**Authors:** Gauraang Bhatnagar, Laura Quinn, Antony Higginson, Andrew Plumb, Steve Halligan, Damian Tolan, Roger Lapham, Susan Mallett, Stuart A. Taylor, METRIC study investigators, Jade Dyer, Pranitha Veeramalla, Sue Tebbs, Steve Hibbert, Richard Ellis, Fergus Thursby-Pelham, Richard Beable, Nicola Gibbons, Claire Ward, Anthony O’Connor, Hannah Lambie, Rachel Hyland, Nigel Scott, Doris Quartey, Deborah Scrimshaw, Helen Bungay, Maggie Betts, Simona Fourie, Rajapandian Ilangovan, Uday Patel, Evgenia Mainta, Phillip Lung, Ian Johnston, Mani Naghibi, Morgan Moorghen, Adriana Martinez, Francois Porte, Christopher Alexakis, James Pilcher, Anisur Rahman, Jonny Vlahos, Rebecca Greenhalgh, Anita Wale, Teresita Beeston, Wivijin Piga, Joey Clemente, Farooq Rahman, Simona de Caro, Shameer Mehta, Roser Vega, Roman Jastrub, Harbir Sidhu, Hameed Rafiee, Mairead Tennent, Caron Innes, Craig Mowat, Gillian Duncan, Steve Morris

**Affiliations:** 1 Centre for Medical Imaging, University College London, London, UK; 2 Institute of Applied Health Sciences, College of Medical and Dental Sciences, University of Birmingham, Birmingham, UK; 3 Department of Radiology, Queen Alexandra Hospital, Portsmouth Hospitals NHS Trust, Portsmouth, UK; 4 Department of Radiology, St James’ University Hospital, Leeds Teaching Hospitals NHS Trust, Leeds, UK; 5 UCL Centre for Medical Imaging, 2nd Floor Charles Bell House, 43-45 Foley Street, London, W1W 7TS UK

**Keywords:** Crohn disease, Ultrasonography, Observer variation, Prospective studies

## Abstract

**Purpose:**

To prospectively evaluate interobserver agreement for small bowel ultrasound (SBUS) in newly diagnosed and relapsing Crohn’s disease.

**Methods:**

A subset of patients recruited to a prospective trial comparing the diagnostic accuracy of MR enterography and SBUS underwent a second SBUS performed by one of a pool of six practitioners, who recorded the presence, activity and location of small bowel and colonic disease. Detailed segmental mural and extra-mural observations were also scored. Interobserver variability was expressed as percentage agreement with a construct reference standard, split by patient cohort, grouping disease as present or absent. Prevalence adjusted bias adjusted kappa (PABAK), and simple percentage agreement between practitioners, irrespective of the reference standard, were calculated.

**Results:**

Thirty-eight patients (11 new diagnosis, 27 relapse) were recruited from two sites. Overall percentage agreement for small bowel disease presence against the consensus reference was 82% (52–95% (95%CI)), kappa coefficient (*κ*) 0.64, (substantial agreement) for new diagnosis and 81%, *κ* 0.63 (substantial agreement) for the relapsing cohort. Agreement for colonic disease presence was 64%, *κ* 0.27 (fair agreement) in new diagnosis and 78%,*κ* 0.56 (moderate agreement) in the relapsing cohort. Simple agreement between practitioners was 84% and 87% for small bowel and colonic disease presence respectively. Practitioners agreed on small bowel disease activity in 24/27 (89%) where both identified disease. Kappa agreement for detailed mural observations ranged from *κ* 0.00 to 1.00.

**Conclusion:**

There is substantial practitioner agreement for small bowel disease presence in newly diagnosed and relapsing CD patients, supporting wider dissemination of enteric US.

**Electronic supplementary material:**

The online version of this article (10.1007/s00261-020-02405-w) contains supplementary material, which is available to authorized users.

## Introduction

Meta-analyses suggest that small bowel ultrasound (SBUS) achieves a high sensitivity for the presence and extent of small bowel Crohn’s disease (CD), recently confirmed in a prospective multicentre trial setting by the METRIC trial [1–4]. SBUS has several advantages over Magnetic resonance enterography (MRE). It does not require oral or intravenous contrast and is preferred by patients [5]. Furthermore, it is widely available and can be employed at both bedside and out-patient clinic [6].

SBUS uptake has been hampered by perceptions of high levels of operator dependence i.e. inter-observer variability. While sonographic features of advanced CD, such as mural thickening and increased colour doppler flow, are usually appreciated readily [7–10], subtle disease can be difficult to differentiate from normal bowel. Segmental localisation is also technically challenging given small bowel length, configuration and motility.

Few data support assertions that SBUS suffers from greater inter-observer variability than any other imaging modality employed for CD. In reality, most studies have concentrated on agreement for morphological signs such as wall thickening, and complications such as strictures and abscess [11, 12]. There is very little research investigating inter-observer variability simply for disease presence, but this is arguably the most important consideration for patient management. Indeed, when it has been investigated, Parente et al. reported substantial agreement between investigators for correct segmental localisation of CD lesions [13].

A proportion of patients recruited to the METRIC trial [4] underwent repeat SBUS by a different practitioner specifically to assess inter-observer variability for detection, extent and descriptive features of small bowel and colonic CD. Our findings are reported here.

## Methods

### Study population

METRIC was a multicentre, prospective cohort trial comparing diagnostic accuracy of MRE and SBUS for the presence, extent and activity of enteric Crohn’s disease [4, 14]. The trial recruited two patient cohorts: (1) newly diagnosed and (2) established disease, clinically suspected of luminal relapse. Patients were eligible for the new diagnosis subgroup if they had been diagnosed with Crohn’s disease in the 3 months preceding recruitment based on conventional diagnostic criteria, or where Crohn’s disease was strongly suspected based on imaging or endoscopic features but pending final diagnosis. Patients were eligible for the suspected luminal relapse subgroup if they had established Crohn’s disease (for greater than 3 months) and high clinical suspicion of luminal relapse based on objective markers of inflammatory activity (CRP > 8 mg/l or faecal calprotectin > 100mcg/g), and/or symptoms suggestive of luminal stenosis (including obstructive symptoms such as colicky abdominal pain, vomiting), and/or abnormal endoscopy. Eligible patients for both arms were aged ≥ 16. Patients were ineligible if pregnant or if they had contraindications to MRI. Full ethical permission was obtained (13/09/2013, REC ref 13/SC/0394), and all patients gave written consent prior to participation.

### Study design

There were eight recruitment sites, two of which agreed to participate in the current study. These asked recruits to undergo a second SBUS, performed by a different practitioner. Additional written consent was taken, and a maximum 7 days was permissible between the two examinations.

Six practitioners (five radiologists and one sonographer) performed and interpreted SBUS for the current reproducibility study (for experience see online resource, table S1). All radiologists were affiliated with the British Society of Gastrointestinal and Abdominal Radiology (BSGAR) with declared subspecialty interest in gastrointestinal radiology [4]. The sonographer had undergone formal training according to their sites’ local polices and was performing SBUS routinely [4].

Patients were fasted for 4 to 6 h. No oral or intravenous contrast was used. Practitioners were blinded to findings from the prior SBUS, and to all other imaging, endoscopic and clinical data other than the cohort from which the patient was recruited (i.e. new diagnosis or relapse), and surgical history [4]. Examinations were performed using standard equipment (either Acuson S3000 US system, Siemens Medical Solutions USA, CA, USA or E Logiq E9, GE Medical Systems Ltd, Buckinghamshire, UK) using both low and high frequency probes, and both grey-scale and colour/power Doppler modes.

For each patient, practitioners completed a case report form (CRF) (see online resource appendix 1), documenting the presence and extent of small bowel and colonic CD using conventional criteria [15]. They were, however, instructed specifically to interpret the examination as they would in routine clinical practice. The small bowel and colon were divided into 4 and 6 segments respectively (see online resource, appendix 2). Practitioners documented their diagnostic confidence for disease presence from 1 to 6, 6 being greatest confidence. Specifically, for disease presence, practitioners scored 1—disease definitely not present, 2—disease probably not present, 3—disease possibly not present, 4—disease possibly present, 5—disease probably present, 6—disease definitely present. The CRF specifically grouped confidence levels 1 and 2 as normal, 3 and 4 as equivocal and 5 and 6 as abnormal.

For those segments scoring 3 or more for disease presence, practitioners also categorised several observations detailing mural and extra-mural appearances (for example wall thickening, mesenteric fat echogenicity, submucosal layer thickening: (see supplementary appendix 3) and stated if, in their opinion, disease was active or not, again using confidence scores of 1 to 6. For disease activity, practitioners scored 1—disease definitely not active, 2—disease probably not active, 3—disease possibly not active, 4—disease possibly active, 5—disease probably active, 6—disease definitely active. Suggested criteria for active disease were provided as part of the main METRIC protocol (wall thickening, focal hyperechoic mesentery (with or without fat wrap), isolated thickened submucosal layer, poorly defined anti-mesenteric border, increased doppler vascular pattern, ulceration or abscess).

A laminated key containing example images for all recorded observations was provided for reference during SBUS interpretation (see online resource appendix 3) Practitioners also recorded the length of disease, the presence of stenosis, and any extra-enteric complications such as abscess or fistulae.

### Reference standard

The reference standard for disease presence, extent and activity for the current study was as per the METRIC trial [4], i.e. an outcome-based, construct reference standard (see online resource, appendix 4).

### Statistical analysis

The 6 point confidence scale for disease presence was dichotomised into “no disease” (confidence levels 1 and 2) or “disease present” (confidence levels 3 to 6), mirroring the METRIC trial analysis [4]. The grouping of equivocal findings (confidence level 3 and 4) with positive findings (confidence levels 5 and 6) was pre-specified in the METRIC trial, and reflected the potential impact of equivocal findings on patient management. Disease activity was similarly dichotomised.

Interobserver variability for disease presence and activity was assessed after grouping the data as positive or negative for disease presence and activity according to the consensus reference and expressed as percentage agreement on a per patient level. For disease extent, practitioners had to agree both on disease presence and segmental location. Agreement for disease activity was undertaken for all patients and all segments regardless of agreement on disease presence. Prevalence adjusted bias adjusted kappa (PABAK) was also calculated. Analysis was performed after splitting the cohort into newly diagnosed or suspected relapse and repeated for small bowel and colon separately. Colonic segments were grouped into “right colon” (caecum, ascending and transverse colonic segments), and the “left colon” (descending, sigmoid and rectal segments). Kappa statistics were interpreted as follows: 0.01–0.20 (slight agreement), 0.21–0.40 (fair agreement), 0.41–0.60 (moderate agreement), 0.61–0.80 (substantial agreement) and 0.81–0.99 (almost perfect agreement) [16].

Segmental agreement was displayed graphically. Descriptive statistics for agreement between practitioners irrespective of concordance with the reference standard was also calculated.

For extra-enteric findings where reference standard data were unavailable (free fluid and lymphadenopathy) agreement between radiologists was based on whether one or both radiologists reported the complication (agreement occurring only in the latter case).

Agreement for the descriptive mural and extra-mural appearances was restricted to segments where both practitioners agreed on disease presence. Percentage agreement was used for categorical descriptions. For continuous descriptions, agreement was expressed as the difference between practitioner measurements (mean and standard deviation or median with interquartile range as appropriate). Where more than one segment was diseased, wall thickness was calculated as the mean across disease segments in a patient.

Statistical analysis was performed using STATA 14.2 (STATACorp LLC, Texas USA).

A small proportion of the results (appropriately acknowledged) have been previously published in Health Technology Assessment [17]. The current report represents a more detailed description of the study findings.

## Results

### Demographic data

Forty-three patients were recruited. Of these, five patients in the new diagnosis cohort were withdrawn because they ultimately did not have CD. The study population therefore consisted of thirty-eight patients (11 new-diagnosis and 26 relapse) (see online resource, table S2), representing 23% of the 163 patients recruited to the sites as part of the main METRIC trial. Both SBUS studies were undertaken on the same day in all patients.

### Presentation of results

For all results, “practitioner 1” is the individual who performed the first SBUS for a particular patient, and “practitioner 2” is the individual who performed the subsequent SBUS.

### Small bowel disease presence

Overall, only 4 of 76 (5%) practitioner scores were rated as equivocal (confidence scores 3 and 4), with the rest being ether negative (scores 1 or 2) or positive (scores 5 and 6). All 11 patients in the new diagnosis cohort had small bowel disease by reference standard. Practitioners agreed on disease presence/absence in 10 of these 11 (91%) patients, agreeing (correctly) that disease was present in 9, and agreeing (incorrectly) that disease was absent in 1 patient. They disagreed on disease presence in 1 patient (positive by reference) (Table 1). Overall agreement for disease presence against the consensus reference was 82% (95% CI 52–95%) with a kappa coefficient (*κ*) of 0.64, indicating “substantial” agreement (Table 2). There was 64% (95%CI 35 to 85%) agreement for disease extent (incorporating segmental location matching) against the consensus reference, with *κ* of 0.27 indicating “fair” agreement (Table 2).

**Table 1:** Practitioner agreement on disease presence in the small bowel, right and left colon with reference to the consensus reference standard findings

Small bowel	New diagnosis (DP; DN) Total 11 cases	Relapse (DP; DN) Total 27 cases
Two practitioners agree disease present	9 (9; 0)	18 (18; 0)
Two practitioners agree disease not present	1 (1; 0)	5 (1; 4)
Two practitioners disagree	1 (1; 0)	4 (0; 4)
Colon		
Two practitioners agree disease present	4 (4; 0)	13 (12; 1)
Two practitioners agree disease not present	6 (3; 3)	10 (1; 9)
Two practitioners disagree	1 (1; 0)	4 (2; 2)

*DP* disease positive, *DN* disease negative

**Table 2:** Per patient Interobserver variability for the presence of small bowel Crohn’s disease against the consensus reference

	Newly diagnosis (*N* = 11)						Suspected relapse (*N* = 27)					
	Disease positive^a^ *N* = 11			Disease negative^a^ *N* = 0			Disease positive^a^ *N* = 19			Disease negative^a^ *N* = 8		
	P1 (*n*)	P2 (*n*)	% Positive agreement (95% CI)	% Negative agreement (95% CI)	% Overall agree*	*κ*	P1 (*n*)	P2 (*n*)	% Positive agreement (95% CI)	% Negative agreement (95% CI)	% Overall agreement*	*κ*
Small bowel disease presence	9	10	82 (52 to 95)	–	82	0.64	18	18	95 (75 to 99)	50 (22 to 78)	81	0.63
Small bowel disease extent^b^	7	9	64 (35 to 85)	–	64	0.27	14	14	58 (36 to 77)	50 (22 to 78)	56	0.11

Reproduced with permission from Taylor et al. [17] Contains information licensed under the Non-Commercial Government Licence v2.0

*P1* number of positive reads practitioner 1, *P2* number of positive reads practitioner 2

^a^Patient classification by consensus reference standard

^b^Small bowel disease extent—Disease presence and correct location in the small bowel

*Both practitioners agree with consensus reference standard

Nineteen of 27 patients (70%) in the relapse cohort had small bowel disease by reference standard. Both practitioners agreed on disease presence in 18 of the 19 disease positive patients (Table 1), and (incorrectly) agreed that disease was absent in 1 patient. Of the 8 patients without small bowel disease, the two practitioners agreed that disease was absent in 4 and disagreed in 4 (Table 1). Overall agreement for disease presence against the reference was 81% with a *κ* of 0.63 indicating “substantial” agreement (Table 2). Agreement for disease extent was 58%, with a *κ* of 0.11 indicating “slight” agreement (Table 2).

### Agreement according to small bowel segment

**Fig. 1:**
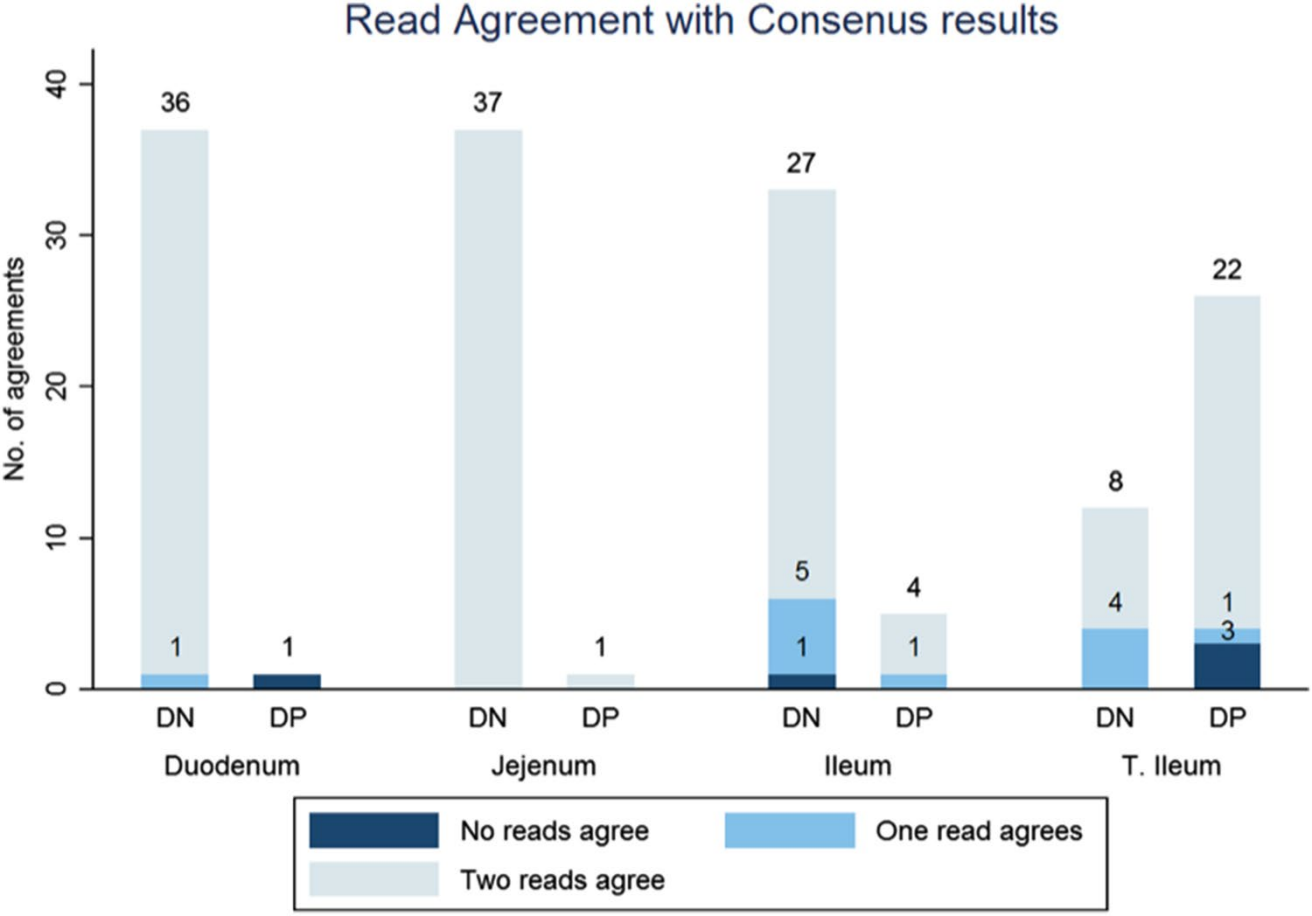
Presence of small bowel Crohn’s disease: Agreement between two reads and consensus reference. Number of patients are shown by segment and disease status (*DP* disease positive, *DN* disease negative), where two reads (dark blue), one read (light blue) and none of the reads agree (pale blue) with the consensus

Figure 1 demonstrates segmental agreement between practitioners regarding disease presence (vs. the reference standard), for both patient cohorts combined. The terminal ileum (TI) accounted for 26 of 33 diseased small bowel segments and both practitioners agreed in 22/26 (85%). There were 3 patients where neither practitioner diagnosed TI disease, and a single patient in whom one correctly diagnosed disease. Of 12 patients without TI disease, practitioners agreed in 8 and disagreed in 4.

**Fig. 2:**
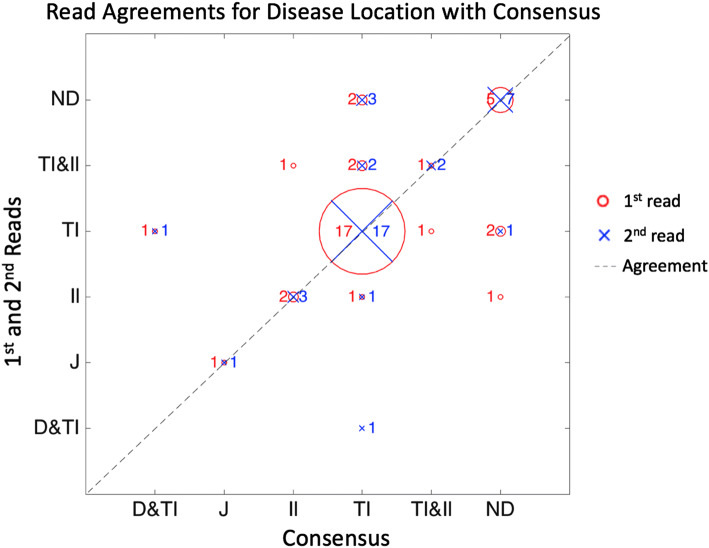
Agreement of first and second reads for disease location compared to consensus reference. The 1st read is shown in red with a circle symbol and the number of patients at the disease location. The 2nd read is shown in blue, using a cross symbol. The diagonal line indicates where reads agree with the consensus. For example, three patients were found to have disease in the Ileum (Il) by the consensus reference. The 1st read agreed with the consensus for two patients (red circle on diagonal numbered 2) and identified one patient with Terminal Ileum (TI) & Ileum (Il) (red circle numbered 1). The 2nd read in blue agreed with the consensus for all three patients (blue cross on diagonal numbered 3). Disease presence: *D&TI* Duodenum & Terminal Ileum, *J* Jejunum, *Il* Ileum, *TI* Terminal Ileum, *TI&Il* Terminal Ileum and Ileum, *ND* Disease Negative

Figure 2 demonstrates agreement for extent of small bowel disease, i.e. presence and segmental localisation (vs. the reference standard). The commonest disease pattern was isolated TI disease (24 of 38 patients) for which both practitioners agreed correctly in 17/24 (71%), and (incorrectly) agreed there was both ileal and terminal ileal disease in 2 patients and isolated ileal disease alone (1 patient).

Eight patients had no small bowel disease by reference standard; practitioner 1 agreed in 5 patients and practitioner 2 in 7 patients.

### Colonic disease

Eight of 11 new diagnosis patients had colonic disease by reference standard. Of these 8 patients, both practitioners incorrectly agreed that there was no disease in 3 patients and disagreed about disease presence in 1 patient (Table 1). Both practitioners agreed correctly there was no disease in all 3 patients who were truly disease negative (Table 1). Overall percentage agreement for disease presence by reference was 64% with a *κ* of 0.27 suggesting “fair” agreement (Table 3). Agreement for disease extent against reference was just 36% with a *κ* of 0.27, again suggesting “fair” agreement (Table 3).

**Table 3:** Per patient Interobserver variability for the presence of colonic Crohn’s disease against the consensus reference

	Newly diagnosed *N* = 11						Suspected relapse *N* = 27					
	Disease positive^a^ *N* = 8			Disease negative^a^ *N* = 3			Disease positive^a^ *N* = 15			Disease negative^a^ *N* = 12		
	P1 (*n*)	P2 (*n*)	% Positive agree (95% CI)	% Negative agree (95% CI)	% Overall agree*	*κ*	R1 (*n*)	R2 (*n*)	% Positive agree (95% CI)	% Negative agree (95% CI)	% Overall agree*	*κ*
Colonic disease presence	5	4	50 (22 to 78)	100 (44 to 100)	64	0.27	14	12	80 (55 to 93)	75 (47 to 91)	78	0.56
Colonic disease extent^b^	2	1	13 (2 to 47)	100 (44 to 100)	36	− 0.27	5	5	13 (4 to 38)	75 (47 to 91)	41	− 0.19

Reproduced with permission from Taylor et al. [17] Contains information licensed under the Non-Commercial Government Licence v2.0

*P1* number of positive reads practitioner 1, *P2* number of positive reads practitioner 2

^a^Patient classification by consensus reference standard

^b^Colonic disease extent—Disease presence and correct location in the small bowel

*Both practitioners agree with consensus reference standard

Fifteen of 27 relapse patients had colonic disease by reference standard. Of these 15 patients, both practitioners agreed correctly that there was disease in 12, agreed (incorrectly) that there was no disease in 1 patient and disagreed regarding disease presence in 2 patients (Table 1). Both practitioners agreed regarding absence of colonic disease in 9 of the 12 (75%) patients without colonic disease.

The overall percentage agreement for disease presence against reference was 78% with a *κ* of 0.56, suggesting “moderate” agreement (Table 3). Agreement for disease extent against reference was 41% with, a *κ* of − 0.19 suggesting no agreement (Table 3).

### Disease activity

Agreement between practitioners and reference standard for per-patient disease activity (irrespective of agreement for disease presence) is shown in Table 4. Agreement was “fair” for the small bowel and right colon but “substantial” for the left colon in newly diagnosed patients.

**Table 4:** Per patient Interobserver variability for the activity of Crohn’s disease in the small bowel, right and left colon against the consensus reference

	New diagnosis *N* = 11								Suspected relapse *N* = 27							
	Disease active^a^				Disease inactive^a^				Disease active^a^				Disease inactive^a^			
	DA (*n*)	P1 (*n*)	P2 (*n*)	% Positive agree (95% CI)	DI (*n*)	% Negative agree (95% CI)	% Overall Agree*	*κ*	DA (*n*)	P1 (*n*)	P2 (*n*)	% Positive agree (95% CI)	DI (*n*)	% Negative agree (95% CI)	% Overall agree*	*κ*
Small bowel	9	7	8	78 (45 to 94)	2	0 (0 to 66)	64	0.27	16	15	12	75 (51 to 90)	11	55 (28 to 97)	67	0.33
Right colon	7	4	4	57 (25 to 84)	4	75 (30 to 95)	64	0.27	17	7	5	56 (21 to 81)	9	76 (53 to 90)	67	0.33
Left colon	7	2	2	50 (15 to 85)	4	100 (65 to 100)	82	0.64	15	10	6	50 (25 to 75)	12	67 (42 to 85)	59	0.19

*DA* disease active by consensus reference, *DI* disease inactive by consensus reference, *P1* number of positive reads practitioner 1, *P2* number of positive reads practitioner 2

^a^Patient activity classification by consensus reference standard

*Both practitioners agree with consensus reference standard

Considering just those segments identified as diseased by both practitioners, (see online resource, table S3), in the 9 new diagnosis patients with small bowel disease, practitioners agreed on disease activity in all 9 (correctly in 7 patients and incorrectly in 2). In the 18 relapse patients with small bowel disease, practitioners agreed on disease activity in 14 (correctly in 12 and incorrectly in 2), agreed on disease inactivity in 1 (correctly), and disagreed on disease activity in 3 (in whom disease was active).

In 6 new diagnosis patients diagnosed with colonic disease by both practitioners, there was agreement for activity in all 6. In the 15 relapse patients diagnosed with colonic disease by both practitioners, there was agreement for activity in 11 (see online resource, table S3).

When considering activity at a segmental level, when both practitioners had agreed regarding disease presence, there was “substantial” agreement for the small bowel (agreement 86% (69 to 94% (95%CI), *κ* 0.71) and near perfect colonic agreement (agreement 93% (81 to 97% (95%CI), *κ* 0.85) (Table 5).

**Table 5:** Interobserver variability for disease descriptions in segments where both practitioners agreed on disease presence

Disease descriptions	Categories in disease descriptions	Small bowel segments *N* = 28		Colon segments *N* = 41	
		% Overall agree (95% CI)	*κ*	% Overall agree (95% CI)	*κ*
Wall thickening	4	54 (36 to 70)	0.07	41 (28 to 57)	0.17
Stenosis causing functional obstruction	2	61 (42 to 76)	0.21	100 (91 to 100)	1.00
Mesenteric fat echogenicity	5	39 (24 to 58)	0.21	44 (30 to 59)	0.12
Anti-mesenteric border	2	61 (42 to 76)	0.21	88 (74 to 95)	0.76
Mesenteric border	3	61 (42 to 76)	0.21	59 (43 to 72)	0.17
Submucosal layer thickness	2	96 (82 to 99)	0.93	100 (91 to 100)	1.00
Submucosal layer echogenicity	4	50 (33 to 67)	0.00	54 (39 to 68)	0.07
Submucosal layer clarity	2	39 (24 to 58)	0.21	61 (46 to 74)	0.22
Mucosal layer thickness	3	54 (36 to 70)	0.07	20 (10 to 34)	0.61
Ulceration	3	54 (36 to 70)	0.07	61 (46 to 74)	0.22
Doppler vascular pattern axial section	3	43 (27 to 61)	0.14	39 (26 to 54)	0.22
Peristatic distension	2	61 (42 to 76)	0.21	71 (56 to 82)	0.41
Segment contains established fibrosis	2	57 (39 to 73)	0.14	85 (72 to 93)	0.71
Segmental disease severity assessment	3	46 (30 to 64)	0.07	44 (30 to 59)	0.12
Segment shows active disease	2	86 (69 to 94)	0.71	93 (81 to 97)	0.85

### Extraluminal complications

Although numbers with extraluminal disease were small (3 patients with abscess and 1 with fistula), there was “almost perfect” agreement (overall agreement across all patients of 97%, *κ* of 0.95) for abscess diagnosis (although this was only diagnosed in 3 patients) and “almost perfect” agreement (overall agreement 95%, *κ* 0.89) for diagnosis of fistula (diagnosed in 1 patient) (see online resource, table S4).

### Detailed segmental disease characteristics

Agreement was “almost perfect” for submucosal layer thickness for both small bowel and colon (Table 5). Agreement was “substantial” for segmental disease activity, appearance of the colonic anti-mesenteric border, and for suspected colonic fibrosis. Agreement for the majority of other variables was “slight” to “fair” (Table 5).

The mean difference in measured wall thickness between the 2 practitioners was 1.6 mm (SD 1.5 mm) for small bowel and 1.5 mm (SD 1.0 mm) for colon. Median difference in length of abnormal bowel was 4 cm (inter-quartile range 2 to 11 cm) and 7 cm (IQR 5 to 10 cm) for small bowel segments and colon segments respectively (see online resource, table S5, Figure S1, S2).

## Discussion

We report substantial sonographic agreement for the presence of small bowel CD, both in newly diagnosed patients and those suspected of luminal relapse. Agreement for colonic disease presence was substantial in the relapse cohort and fair for new diagnoses. Agreement for small bowel and colonic disease extent (i.e. presence and segmental location) was inferior to that for disease presence alone.

Our primary analysis compared practitioner agreement with the outcome-based consensus reference standard used in the METRIC trial rather than with each other because high levels of inter-observer agreement in the face of low diagnostic accuracy has no clinical utility. The METRIC trial found that sensitivity of SBUS for small bowel disease presence and extent were 92% and 70% respectively [4]. Our primary analysis therefore incorporates the intrinsic diagnostic accuracy of SBUS for Crohn’s disease, and provides a more realistic reflection of clinical utility.

However, we did analyse agreement independent of the reference standard, i.e. how often did practitioners agree with each other, even if wrong. In this regard, practitioners agreed regarding presence of small bowel disease in 89% of patients. Furthermore, when analysed at a segmental level, agreement remained high suggesting that a sizeable proportion of the disagreement between practitioner pairs and the reference standard was driven by the limitations of SBUS itself, i.e. different practitioners tend to miss the same disease, presumably due to the subtlety of findings, uncommon morphology, and/or problems with visualisation due to body habitus or disease location.

Agreement for small bowel disease extent was lower than simply for disease presence. Our results for disease extent are somewhat at odds with those of Parente et al. who reported near perfect agreement for segmental localisation (*κ* 0.91) between two experienced investigators in 102 patients [13]. Unlike the present study, Parente utilised just two highly experienced observers and a softer reference standard, in part based on barium fluoroscopy.

In general, results for the colon were similar to small bowel, with relatively good agreement between practitioners for colonic disease presence, but poor agreement for extent.

Against reference standard, we found only fair agreement for assessment of disease activity on a per-patient basis, but when we restricted our analysis to segments identified correctly by both practitioners, we reassuringly found “substantial” and “near perfect” agreement for small bowel and colonic activity respectively, suggesting that once disease is diagnosed, agreement for underlying activity is generally acceptable.

Our patients had very few extraluminal complications, although when present, they tended to be detected by both practitioners. Fraquelli et al., reported variability for diagnosis of fistula and abscess (with *κ* ranging from 0.31 to 1) [11], although whereas Dillman et al. demonstrated near perfect agreement (*κ* 0.96) for abscess diagnosis in paediatric patients using methodology similar to ours [12].

We also investigated agreement for several enteric and extra-enteric sonographic observations associated with CD. One of the most important is bowel wall thickness. We found that agreement was only slight if bowel wall thickness measurements are placed into one of 4 pre-defined categories. When treated as a continuous variable we found a mean difference below 2 mm between practitioner measurements. Although a relatively small numerical difference, this does suggest that using strict cut off measurements for abnormal bowel such as 3 mm should be perhaps be used with caution. Our data are consistent with the findings of Fraquelli et al., who demonstrated substantial to near perfect agreement (with *κ* ranging from 0.72 to 1) for bowel wall thickness measurement [11]. Similarly, Dillman et al., reported substantial correlation or maximum wall thickness measurements (ICC of 0.67) [12]. There was also reasonable agreement for small bowel length measurements, again similar to that reported by Dillman et al. [12].

Agreement for other proposed categorical US descriptors of CD stigmata was variable, even when an image key is used. However, the main clinical utility of SBUS is to detect disease and assess activity and, as discussed above, agreement was reasonable in this regard. We also found fair agreement for diagnosis of small bowel stenosis and perfect agreement for the colon. Fraqueli et al. demonstrated substantial to near perfect agreement and Dillman et al. reported moderate agreement for strictures [11, 12].

Our study does have limitations. Although 8 recruitment sites participated in METRIC, only 2 (with 6 practitioners) participated in this substudy. Our results should therefore be viewed in the context of a relatively small sample of METRIC radiologists. While kappa statistics are used widely to express agreement, they do not always indicate the full clinical implications of findings. We do however report percentage agreement which will be more intuitive for clinicians. As noted, the grouping of equivocal findings (confidence level 3 and 4) with positive findings (confidence levels 5 and 6) was pre-specified in the METRIC trial, and reflected the potential impact of equivocal findings on patient management. Arguably, based on the definitions of the 6 confidence scores, grouping scores 1 to 3 and comparing with scores 4 to 6 would seem reasonable. However, the practitioners were specially told scores 3 and 4 should be considered equivocal when they completed the CRFs. In reality there were very few equivocal scores for disease presence (just 5%), so alternative approaches to handling them in the analysis did not meaningfully impact on the study findings.

In conclusion, in our multicentre prospective cohort study we found substantial agreement between practitioners for the presence of small bowel CD in newly diagnosed patients, and patients with suspected relapse. Agreement for categorising disease as active or otherwise is also high, but agreement for disease extent is slight or fair, reflecting the intrinsic difficulties of attempting to fully interogate small bowel. Sonographic agreement for categorical descriptors of CD stigmata is variable.

## Electronic supplementary material

Below is the link to the electronic supplementary material.

Supplementary material 1 (PDF 1213 kb)
