# Supplementary material for: Observer agreement for small bowel ultrasound in Crohn’s disease: results from the METRIC trial
Source: Abdom Radiol (NY). 2020 Feb 10;45(10):3036–45. doi: 10.1007/s00261-020-02405-w (PMC7455580; doi:10.1007/s00261-020-02405-w)

Supplementary material 1

Table S1 Practitioner experience in small bowel ultrasound

Practitioner	Training	Recruitment site	Overall experience of gastrointestinal Imaging (at start of trial) [years]	Estimated total small bowel US performed (at start of trial)
1	Radiologist	1	3	50
2	Radiologist	1	15	>1000
3	Radiologist	1	7	>500
4	Radiologist	1	20	>1000
5	Radiologist	2	12	50
6	Sonographer	2	20	50

Appendix 1 Case Report Form:

Radiologist Initials_____________

USS platform_____________________

Are you blinded to other clinical tests and investigations and patient clinical history (other than previous surgical history and new diagnosis or relapse cohort) ☐ **Yes** ☐ **No**

If N state what information/ test data you are aware of (eg barium FT)____________________

Scan type (please circle)

Main Metric trial scan   Hydosonography substudy   Reader agreement substudy

If main Metric scan is this a repeat USS due to unblinded first USS examination? ☐ **Yes** ☐ **No**

**Hydrosonography sub study**			
**Performed using oral contrast given for same day MRE (circle)**	☐ **Yes**	☐ **No**	
**Type of oral contrast**		**Volume ingested**	
**Time for ingestion**			

Case Report Form: Ultrasound Scanning: Scan quality

**Segment**	**Quality of segmental visualisation to make correct diagnosis**<br>**1-good/ 2-moderate/ 3-poor/ 4-N/A or excised**				**If poor visualisation, tick why**<br>**1- overlying bowel gas/ *2- increased patient BM/ 3-Difficult anatomy***/ 4-patient pain/ 5-other (state)***				
	1	2	3	4	1	2	3	4	5
**duodenum**									
**Jejunum***									
**ileum**									
**Terminal ileum****									
**Caecum**									
**Ascending**									
**Transverse**									
**Descending**									
**Sigmoid**									
**Rectum**									

* small bowel from DJ flexure mainly to the left of a diagonal running from the RUQ to LLQ showing typical feathery fold pattern,

** last 10cm of ileum upstream of IV valve/anastomosis

***Eg low lying caecum

Ileocaecal valve identified ☐ **Yes** ☐ **No**

Case Report Form: Ultrasound Scanning Disease Assessment

**Overall disease assessment (to be completed for all patients)**						
	**Normal**		**Equivocal**		**Abnormal**	
**Confidence**	1 (disease definitely not present)	2 (disease probably not present)	3 (disease possibly not present)	4 (disease possibly present)	5 (disease probably present)	6 (disease definitely present)
**Any small bowel disease PRESENT?-** **tick confidence box**						
	1 (disease definitely not active)	2 (disease probably not active)	3 (disease possibly not active)	4 (disease possibly active)	5 (disease probably active)	6 (disease definitely active)
**If present (confidence score ≥3) is it ACTIVE? tick confidence box**						
	1 (disease definitely not present)	2 (disease probably not present)	3 (disease possibly not present)	4 (disease possibly present)	5 (disease probably present)	6 (disease definitely present)
**Any colonic disease PRESENT? tick confidence box**						
	1 (disease definitely not active)	2 (disease probably not active)	3 (disease possibly not active)	4 (disease possibly active)	5 (disease probably active)	6 (disease definitely active)
**If present (confidence score ≥3) is it ACTIVE? tick confidence box**						

Case Report Form: Ultrasound Scanning Other findings

**Lymphadenopathy (0-3)**	☐ **0**	☐ **1**	☐ **2**	☐ **3**
**Abnormal free fluid (Y/N)**	☐ **Yes**		☐ **No**	
**Abscess present**	☐ **Yes**		☐ **No**	
If yes please state size & location				
**Fistula present (circle all that apply)**	☐ **Yes**		☐ **No**	
If yes please circle location		Ileo-ilealIleo-colic entero-cutaneous ileo-vesical<br>colon-vesicaljejuno- jejunaljejuno-colic<br>Other (state_______________________________)		
**Other small bowel diagnosis (eg adhesions, meckels, radiation enteritis etc**	☐ **Yes**		☐ **No**	
**If yes please state**				
**Extra enteric findings (eg aortic aneurysm gallstones, solid organ abnormality, phelgmon)**	☐ **Yes**		☐ **No**	
If yes please state				
**Are you recommending any further tests?**	☐ **Yes**		☐ **No**	
If yes please state which				

Case Report Form: Ultrasound Scanning Disease Presence

Please complete for each segment	**Confidence of disease PRESENCE**					
	**Normal**		**Equivocal**		**Abnormal**	
**Segment**	1 (disease definitely not present)	2 (disease probably not present)	3 (disease possibly not present)	4 (disease possibly present)	5 (disease probably present)	6 (disease definitely present)
**Duodenum (D)**						
**Jejunum (J)**						
**Ileum (I)**						
**Terminal ileum (TI)***						
**Caecum (C)**						
**Ascending colon (A)**						
**Transverse colon (T)**						
**Descending colon (D)**						
**Sigmoid (S)**						
**Rectum (R)**						

*throughout, if TI disease is contiguous for over 10cm count just as TI not TI and ileum

Case Report Form: Ultrasound Scanning Disease Activity

Confidence of disease ACTIVITY Please complete for each segmentif confidence scores 3-6 for disease presence above ie **present or equivocal**

		Normal		Equivocal		Active	
**Segment**	No disease (ie confidence scores 1 or 2 for disease presence)	1 (disease definitely not active)	2 (disease probably not active)	3 (disease possibly not active)	4 (disease possibly active)	5 (disease probably active)	6 (disease definitely active)
**Duodenum (D)**							
**Jejunum (J)**							
**Ileum (I)**							
**Terminal ileum (TI)**							
**Caecum (C)**							
**Ascending colon (A)**							
**Transverse colon (T)**							
**Descending colon (D)**							
**Sigmoid (S)**							
**Rectum (R)**							

Case Report Form: Ultrasound Scanning Disease Description Disease Site 1

PLEASE COMPLETE FOR EACH DISEASE SITE (DEFINED AS >3CM OF NORMAL BOWEL BETWEEN DISEASE SITES). USE ONE TABLE FOR EACH DISEASE SITE. ONLY RECORD SEGMENTS WHICH IF YOU HAVE A CONFIDENCE SCORE OF 3 OR MORE FOR DISEASE PRESENCE. USE SCORE DEFINITIONS AT THE START OF THIS CRF

Complete for equivocal or abnormal sites (ie confidence scores 3-6). Complete additional tables as required if multiple disease sites per segment

Location	Tick one location	Single Wall thickness (mm) thickest portion	Wall thickening (0-3)	Length of abnormal bowel (cm)	Stensois causing functional obstruction (0/1)	Mesenteric fat echogenicity	Anti-mesenteric border (0-4)	Mesenteric border (0-2)	Submucosal layer	Submucosal layer echogenicity (0-3)	Submucosal layer clarity (0/1)	Mucosal layer (0-2)	Ulceration (0-2)	Doppler vascular pattern axial section (0-2)	Peristaltic distension (o/1)	Does the segment contain established fibrosis (Y/N)	Segmental disease severity assessment (0-2)	Segment shows active disease (Y/N)
**Duo**																		
**J**																		
**I**																		
**TI**																		
**C**																		
**A**																		
**Des**																		
**S**																		
**R**																		

Appendix 2: Full definitions of bowel segments

duodenum(D), jejunum(J), ileum(IL), terminal ileum(TI) and the colon into rectum(R), sigmoid(S), descending colon(DC), transverse(TC), ascending(AC) and caecum(C).

The J was defined as the proximal bowel lying largely to the left of a diagonal drawn from the right upper quadrant to the left lower quadrant demonstrating a typically feathery fold pattern. The TI (or neo-TI in the case of past resections) was defined as the last 10cm of small bowel upstream of the ileo-colonic junction. Contiguous disease involving the TI but extending beyond 10cm was classified as TI disease (as opposed to both TI and IL). Distinct sections of disease within a particular segment were defined as non-contiguous if 3cm or more of normal appearing small bowel was present between disease sites. Disease sections separated by less than 3cm of normal bowel were considered a single disease section (contiguous) for the purposes of data recording

Appendix 3 Ultrasound Imaging Key

Scoring guide to be used in conjunction with ultrasound CRF pictorial definitions.

**Bowel wall thickness**	
**Score**	
**0**	0-3mm - Normal
**1**	3-6mm
**2**	6-9mm
**3**	>9mm

**Functional obstruction = Upstream dilatation**	
**Score**	
**0**	None
**1**	Present

**Mesenteric fat echogenicity**	
**Score**	
**0**	Normal
**1**	Focal hyperechoic without fat wrap (ie focal defined area of mesenteric fat of increased echogenicity without overall increase in volume of peri mural fat)
**2**	Focal hyperechoic with fat wrap (ie focal defined area of mesenteric fat of increased echogenicity with overall increase in volume of peri mural fat)
**3**	Stratified heterogeneous with fat expansion (chronic disease) - overall increase in volume of peri mural fat with maintained normal mesenteric stratification and no focal hyperechoic area)
**4**	Uniform hypoechoic (ie mesenteric fat of decreased echogenicity

Anti-mesenteric border	
**Score**	
**0**	Well defined (smooth interface between bowel wall and mesenteric fat)
**1**	Ill defined (irregular Interface between bowel wall and mesenteric fat)

Mesenteric border	
**Score**	
**0**	Well defined (smooth interface between bowel wall and mesenteric fat)
**1**	Generally ill defined (generally irregular Interface between bowel wall and mesenteric fat)
**2**	Focally ill defined (focal irregular Interface between bowel wall and mesenteric fat)

Submucosal layer	
**Score**	
**0**	Normal
**1**	Thickened (increased thickness compared to submucosa of normal bowel in the same patient)

Submucosal layer echogenicity	
**Score**	
**0**	Normal
**1**	Reduced (echogenicity lower than normal bowel in same patient)
**2**	Uniform increased (echogenicity greater than normal bowel in same patient)
**3**	Increased with bands (echogenicity greater than normal bowel in same patient with spoke wheel hypoechoic bands)

Submucosal layer clarity	
**Score**	
**0**	Normal - well defined (smooth interface between submucosa and other wall layers)
**1**	Ill defined (irregular interface between submucosa and other wall layers)

Mucosal layer	
**Score**	
**0**	Well defined (smooth interface between bowel wall and mesenteric fat)
**1**	Isolated thickened (increased thickness compared to mucosa of normal bowel in the same patient)
**2**	Thickened also in presence of submucosal thickening

Ulceration	
**Score**	
**0**	None
**1**	Superficial-< 50% wall thickness
**2**	Deep-≥50% wall thickness

Doppler vascular pattern imaged in axial section	
**Score**	
**0**	Well defined (smooth interface between bowel wall and mesenteric fat)
**1**	Increased focal (increased Doppler signal isolated to less than half the circumference on a trans-axial image) compared to normal bowel in the same patient)
**2**	Increased generalised (increased Doppler signal affecting more half the circumference on a trans-axial image compared to normal bowel in the same patient)

Peristalsis related to stricturing	
**Score**	
**0**	Distension of the segment during peristaltic wave
**1**	Non distension of segment during proximal peristaltic wave

Disease activity
**At least one of: wall thickening/ focal hyperechoic mesentery (with or without fat wrap)/ isolated mucosal thickening /ill defined submucosal layer/ ill defined anti-mesenteric border/ Increased Doppler vascular pattern OR ulceration OR abscess**

Segmental disease severity assessment	
**Score**	
**0**	None
**1**	Early-superficial ulceration and/or mild wall thickening/ mild increased vascularity
**2**	Advanced-transmural disease and/or fistulation and/or stricturing and/or cobblestoning/ and or wall oedema or significant thickening

Lymphadenopathy (all measurements in short axis)	
**Score**	
**0**	None
**1**	Cluster all less than 1cm
**2**	1 or 2 nodes > 1cm
**3**	3 or more nodes greater than 1cm

Case Report Form-USS SCORING

Mesenteric Fat Echogenicity

0 - Normal

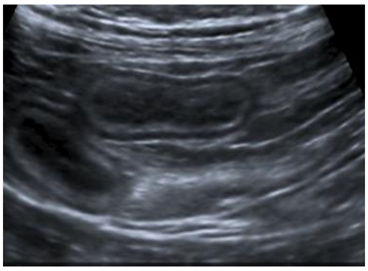

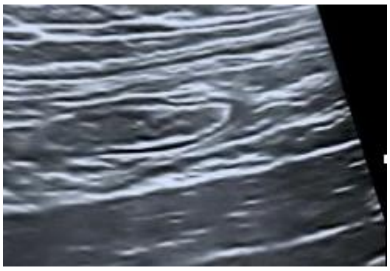

1 - Focal hyperechoic without fat wrap (ie focal defined area of mesenteric fat of increased echogenicity without overall increase in volume of peri mural fat)

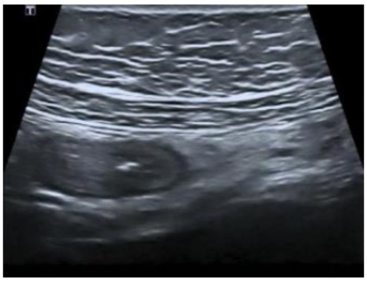

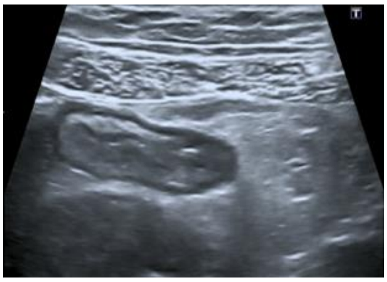

2 - Focal hyperechoic with fat wrap (ie focal defined area of mesenteric fat of increased echogenicity with overall increase in volume of peri mural fat)

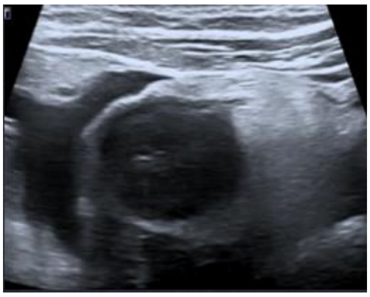

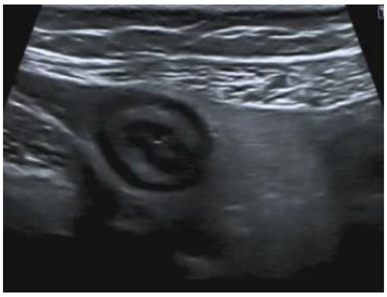

3 - Stratified heterogeneous with fat expansion (chronic disease) - overall increase in volume of peri mural fat with maintained normal mesenteric stratification and no focal hyperechoic area**)**

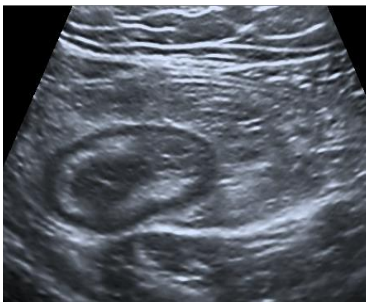

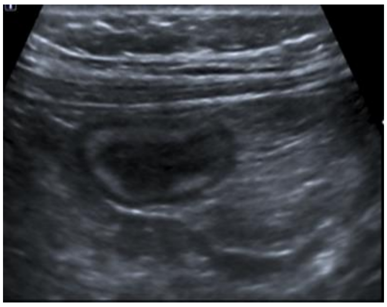

4 - Uniform hypoechoic (ie mesenteric fat of decreased echogenicity

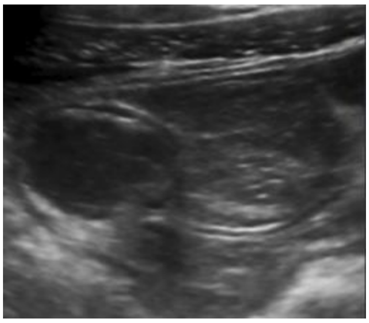

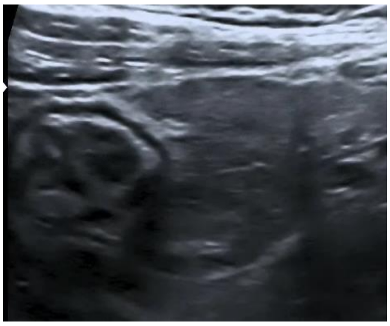

Case Report Form-USS SCORING

Anti-Mesenteric Border

Example 1

0 - Well defined (smooth interface between bowel wall and mesenteric fat)

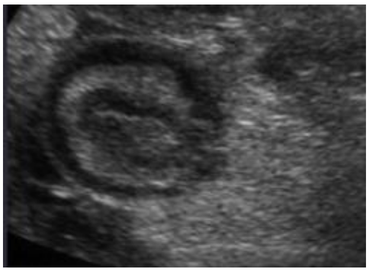

Post treatment

1 - Ill defined (irregular Interface between bowel wall and mesenteric fat)

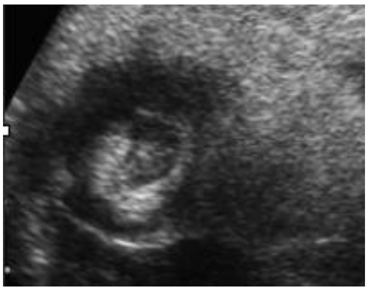

Pre treatment

Example 2

0 - Well defined (smooth interface between bowel wall and mesenteric fat)

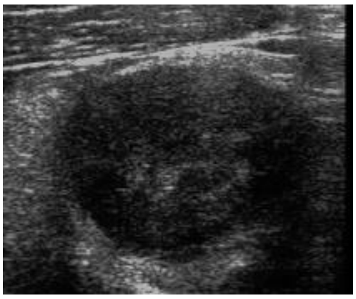

Post treatment

1 – Ill defined (irregular Interface between bowel wall and mesenteric fat

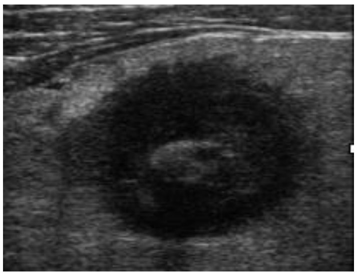

Pre treatment

Case Report Form-USS SCORING

Mesenteric Border

0 - Well defined

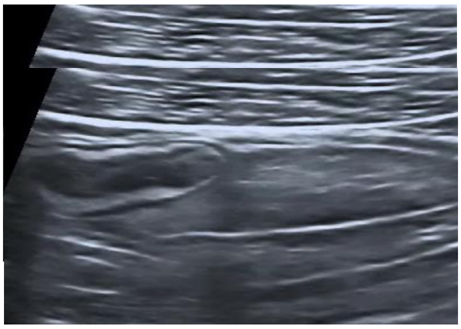

1 - Ill defined

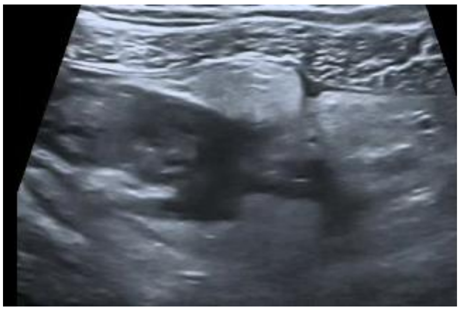

2 - Focal involvement

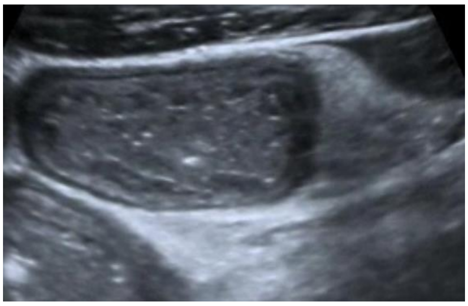

Submucosal layer

Ileostomy

0 - Normal

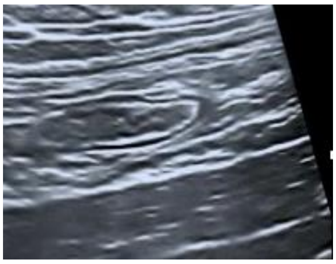

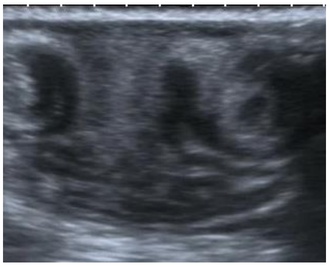

1 - Thickened (increased thickness compared to submucosa of normal bowel in the same patient

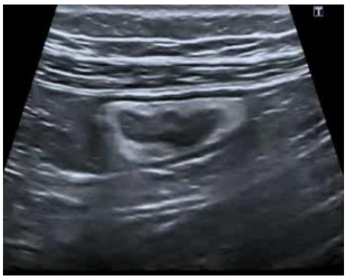

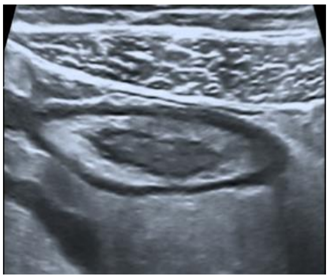

Case Report Form-USS SCORING

Sub-mucosal layer Echogenicity

0 - Normal

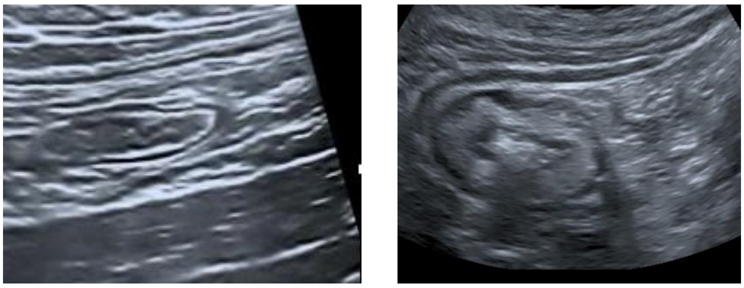

1 - Reduced (echogenicity lower than normal bowel in same patient)

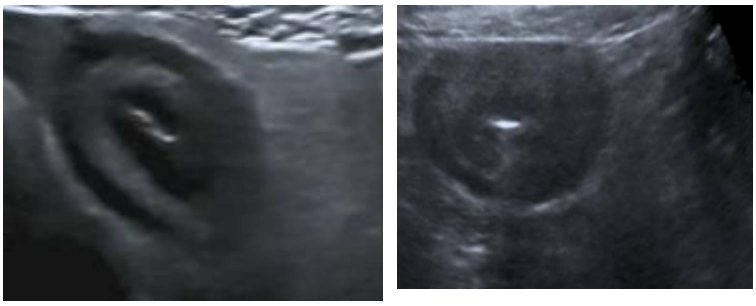

2 - Uniform increased (echogenicity greater than normal bowel in same patient)

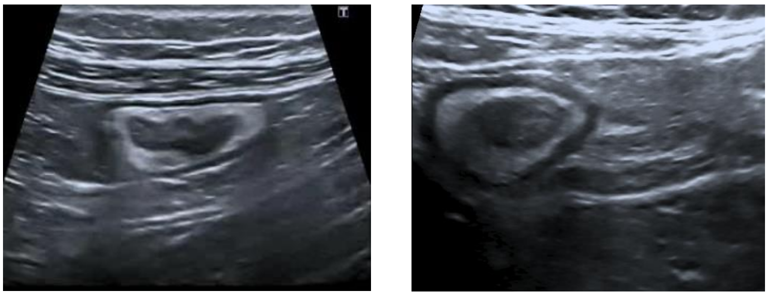

3 - Increased with bands (echogenicity greater than normal bowel in same patient with spoke wheel hypoechoic bands)

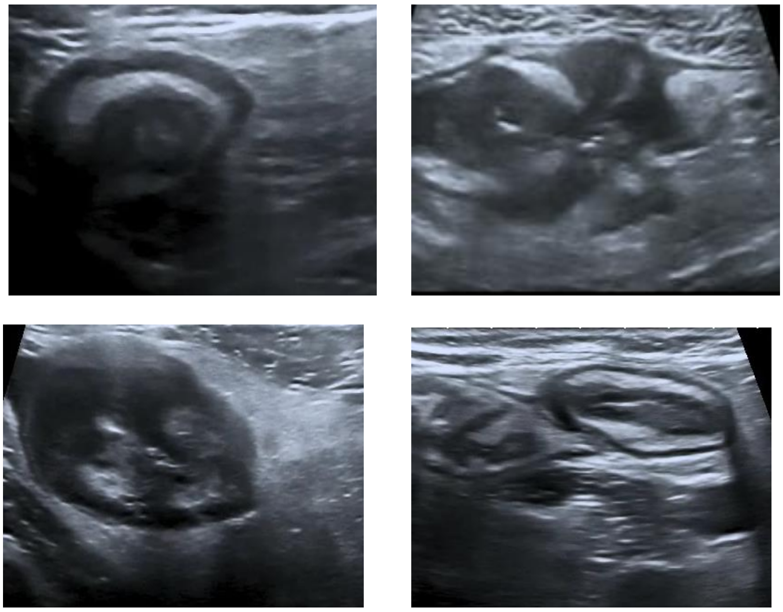

Case Report Form-USS SCORING

Sub-mucosal Layer Clarity

0 - Normal well defined (smooth interface between submucosa and other wall layers)

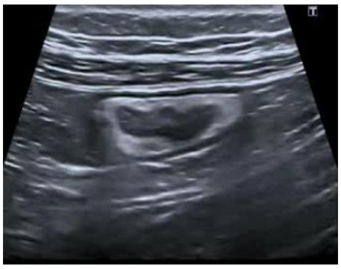
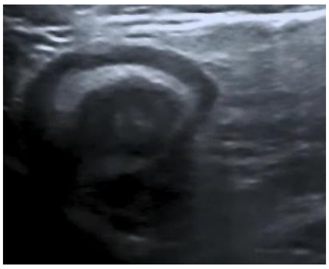

1 - Ill defined (irregular interface between submucosa and other wall layers)

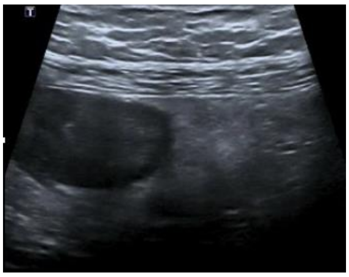
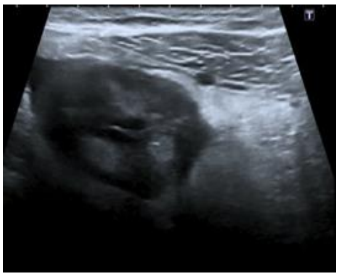

Mucosal layer

0 - Normal

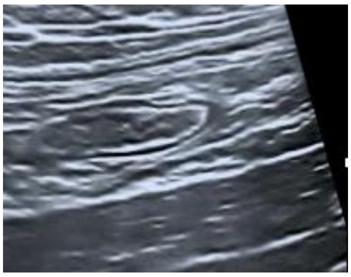
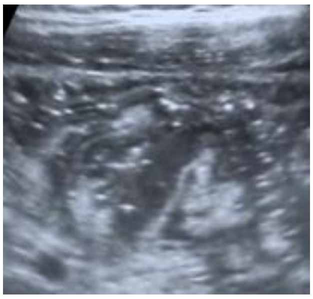

ileocaecal valve ulceration

1 - Isolated thickened (increased thickness compared to mucosa of normal bowel in the same patient

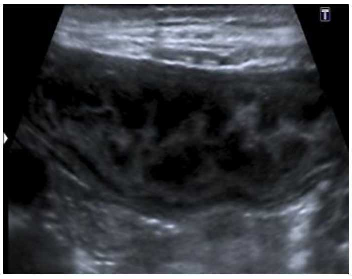
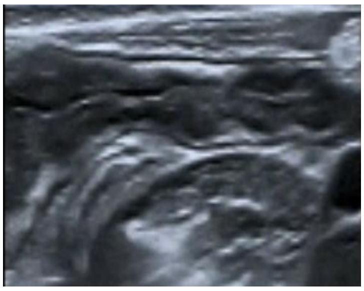

2 - Thickened also in the presence of submucosal thickening

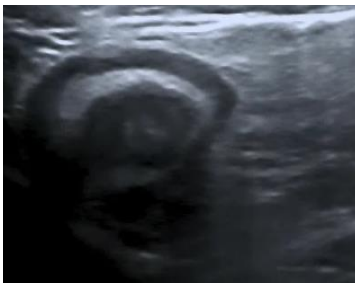
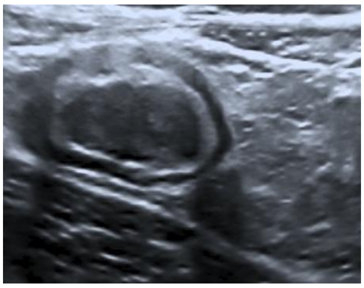

Case Report Form-USS SCORING

Doppler Vascular Pattern Imaging In Axial Section

0 - Normal

1 - Increased focal (increased Doppler signal isolated to less than half the circumference on a trans-axial image) compared to normal bowel in the same patient)

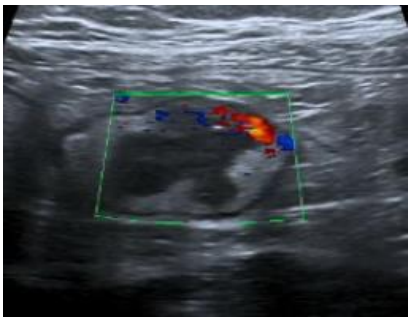

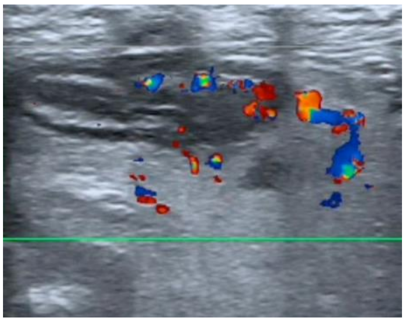

2 - Increased generalised (increased Doppler signal affecting more half the circumference on a trans-axial image compared to normal bowel in the same patient)

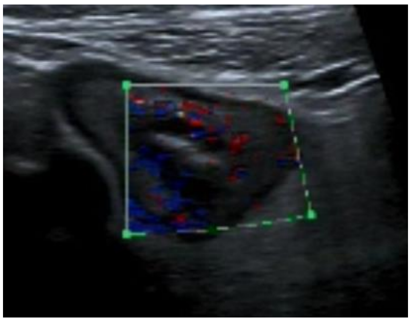

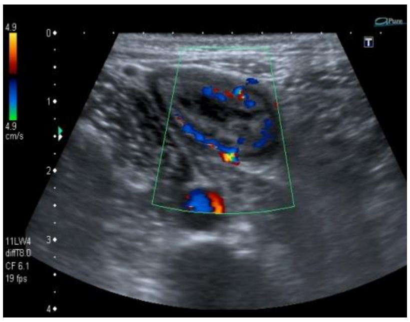

Ulceration

0-Normal

Superficial-< 50% wall thickness

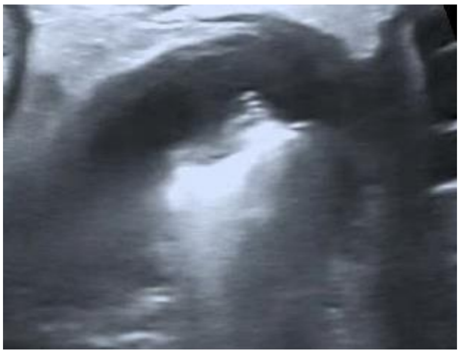

Deep-≥50% wall thickness

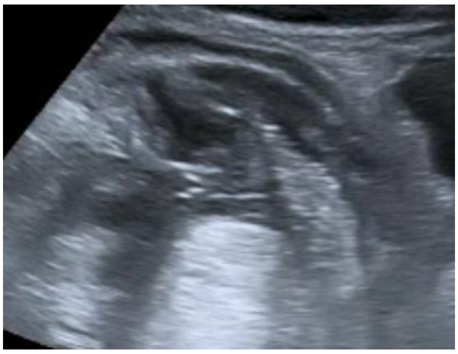

Case Report Form-USS SCORING

Functional obstruction = upstream dilatation

Peristalsis

1-Distension of the segment during peristaltic wave

2-Non distension of segment during proximal peristaltic wave

Disease activity At least one of: wall thickening/ focal hyperechoic mesentery (with or without fat wrap)/ isolated mucosal thickening /ill defined submucosal layer/ ill defined anti-mesenteric border/ Increased Doppler vascular pattern OR ulceration OR abscess

Disease assessment

Early-superficial ulceration and/or mild wall thickening/ mild increased vascularity

Advanced-transmural disease and/or fistulation and/or stricturing and/or cobblestoning / and or wall oedema or significant thickening

Lymphadenopathy

0=none. 1=cluster all less than 1cm. 2=cluster 1 or2 nodes >1cm. 3=3 or more nodes greater than 1cm

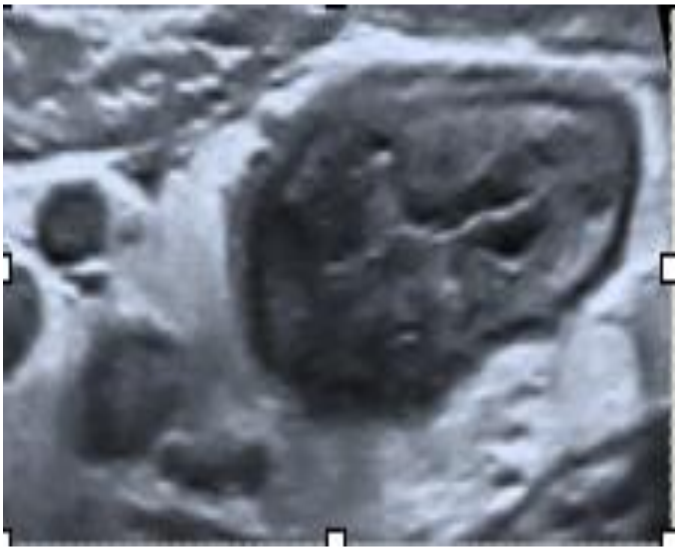

Appendix 4: Reference standard for disease presence in the METRIC trial

Patients were followed for a period of 6 months following recruitment. Each recruitment site then held a series of consensus panels which considered all information collected over the follow-up period including all imaging, endoscopies, surgical findings**,** histopathology, biochemical markers, and clinical course. The panels included at least one local gastroenterologist, one local radiologist and one radiologist from another recruitment site. The panel recorded whether small bowel or colonic Crohn's disease was present, and, if so, whether disease was active. Disease could only be categorised as active if there was at least one endoscopic, biochemical or histopathological objective marker (ulceration as seen at endoscopy, measured CRP concentration >8 mg/L, measured calprotectin concentration >250 µg/g, histopathological evidence of acute inflammation based on a biopsy sample or surgery within 2 months of trial imaging)

Table S2 Characteristics of patients according to patient cohort.

Patient characteristics	New diagnosis [n (%)] N=11	Suspected relapse [n (%)] N=27
**Age - yrs., mean(range)**	37 (21 to 84)	32 (17 to 71)
**Male**	6 (55)	11 (41)
**Previous enteric surgery**		
Yes	0 (0)	11 (41)
No	11 (100)	16 (59)
**Colonoscopy available to consensus reference panel**	11 (100)	7 (26)
**Disease presence**		
Small bowel	11 (100)	19 (70)
Colon	8 (73)	15 (56)
**Disease duration**		
<1 year	NA	1 (3)
1-5 years	NA	11 (41)
6-10 years	NA	4 (15)
>10 years	NA	11 (41)
**Previous disease location (Montreal classification)**		
L1	NA	10 (37)
L2	NA	5 (18)
L3	NA	11 (41)
L4	NA	1 (4)
**Previous disease behaviour (Montreal classification)**		
B1	NA	11 (41)

B1p	NA	1 (3)
B2	NA	11 (41)
B3	NA	4 (15)
**Inclusion criteria for relapse cohort patients**		
Raised CRP>8mg/l	NA	15 (56)
Raised calprotectin>100	NA	6 (22)
Obstructive symptoms	NA	10 (37)
Abnormal endoscopy	NA	0 (0)

Of the 43 patients recruited for the interobserver study, 5 patients withdrawn due to not having Crohn's disease at consensus stage.

NA= not applicable as characteristics are only relevant to relapse patients

Mean and range values were used for continuous measurements. Counts and percentages were used for categorical measurements.

Table S3 Practitioner agreement on disease activity in the small bowel, and segmental (right and left) colon (only when practitioners agree on disease presence) with reference to the consensus reference standard. DA – disease active, DNA – disease not active

	New diagnosis (In cases of DA; DNA)	Relapse (In cases of DA; DNA)
**Small bowel**	Total 9 patients	Total 18 patients
Two practitioners agree disease active	9 (7;2)	14 (12;2)
Two practitioners agree disease inactive	0 (0;0)	1 (0;1)
Two practitioners disagree on activity	0 (0;0)	3 (3;0)
**Right colon**	Total 4 segments	Total 5 segments
Two practitioners agree disease active	4 (4;0)	5 (5;0)
Two practitioners agree disease inactive	0 (0;0)	0 (0;0)
Two practitioners disagree on activity	0 (0;0)	0 (0;0)
**Left colon**	Total 2 segments	Total 10 segments
Two practitioners agree disease active	2 (2;0)	6 (6;0)
Two practitioners agree disease inactive	0 (0;0)	0 (0;0)
Two practitioners disagree on activity	0 (0;0)	4 (3,1)

Table S4 Disease complications. Positive agreement, overall agreement and prevalence-adjusted bias-adjusted Kappa statistic (κ) are presented according to the number of patients with each disease complication.

	All patients N=38								
	Disease positive				Disease negative				
**Disease Complications**	**DP ˆ (n)**	**P1 (n)**	**P2 (n)**	**% Positive Agree (95% CI)**	**DN ˆ (n)**	**% Negative Agreement (95% CI)**	**% Overall Agree**	κ
Consensus results available								
Abscess	3	2	2	67 (21 to 94)	35	100 (90 to 100)	97	0.95
Fistula	1	1	1	100 (21 to 100)	37	95 (82 to 99)	95	0.89
No consensus results								
Lymphadenopathy		7	4	10 (2 to 40)		76 (60 to 87)	76	0.53
Abnormal free fluid		9	11	33 (15 to 58)		70 (53 to 83)	74	0.47

P1 – Number of positive reads practitioner 1, P2 Number of positive reads practitioner 2

ˆ Patient classification by consensus reference standard

Table S5 Difference in length and wall thickness of abnormal bowel: Interobserver variability of ultrasound scans. The average difference in length and thickness reported from both reads of the same patient by two practitioners. Values are only reported where abnormal bowel is identified by both practitioners. Means and standard deviations or median and interquartile ranges are reported.

Average difference	Small bowel segments N=28	Colon segments N=39
Single wall thickness, mean (SD) in mm	1.6 (1.5)	1.5 (1.0)*
Length of abnormal bowel, median (IQR) in cm	4 (2 to 11)	7 (5 to 10)

* Information for one segment is missing

Figure S3 and S4: Graph of ultrasound length and thickness measurements: comparison of two practitioners. Each dot corresponds to a location identified as abnormal bowel. Disease was classified in two groups: Small bowel and Colon. A patient can have up to two dots if disease is present in each of the classified groups. A small amount of random noise was added to the dots for visual display purpose.

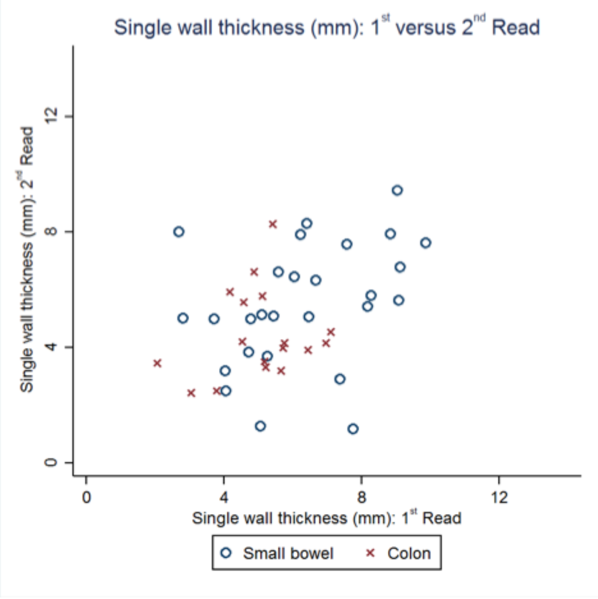

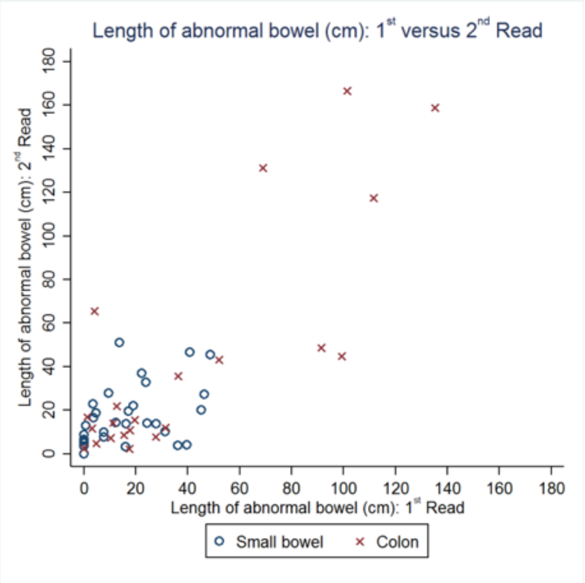

Supplement: Supplementary file 1 — Supplementary material 1 (PDF 1213 kb) [file 261_2020_2405_MOESM1_ESM.pdf]
